# Supplementary material for: On the flexibility of the cellular amination network in E coli
Source: eLife. 2022 Jul 25;11:e77492. doi: 10.7554/eLife.77492 (PMC9436414; doi:10.7554/eLife.77492)
Supplement: Supplementary file 1. — Strains were grown over 170 hr in minimal medium with ammonium (glut-aux) and without ammonium (glut-aux, wildtype). 20 mM glycerol was used as the carbon source and 5 mM amino acid as nitrogen source. Maximal doubling time in a window of 6 hr was calculated using MATLAB and is indicated with standard error. An OD600 of 0.1 was the threshold for defining growth (above OD600 of 0.1) or no growth. [file elife-77492-supp1.docx]

|  | glut-aux (no NH3) | glut-aux (with NH3) | WT  (no NH3) |
| --- | --- | --- | --- |
| Alanine | no growth | no growth | 1.7 ± 0 |
| Valine | no growth | no growth | no growth |
| Leucine | 15.4 ± 0.8 | 13.6 ± 0.2 | 21.8 ± 8.8 |
| Isoleucine | 33.9 ± 1.1 | 26.5 ± 1.1 | no growth |
| Aspartate | 1.9 ± 0 | 1.5 ± 0 | 2.0 ± 0.1 |
| Asparagine | 6.1 ± 0 | 7.3 ± 0 | 2.8 ± 0 |
| Threonine | no growth | no growth | no growth |
| Methionine | 12.9 ± 0.6 | 14.5 ± 0.4 | 17.7 ± 0.8 |
| Lysine | no growth | no growth | 12.5 ± 0.4 |
| β-alanine | no growth | no growth | no growth |
| Histidine | 17.6 ± 0.9 | 26.3 ± 1.9 | no growth |
| Proline | 3.1 ± 0 | 3.1 ± 0 | 3.4 ± 0 |
| Glutamate | 10.3 ± 0.4 | 2.8 ± 0 | 10 ± 0.7 |
| Glutamine | 8.1 +± 0 | 5.2 ± 0.1 | 1.7 ± 0 |
| Ornithine | 4.6 ± 0 | no growth | 5.2 ± 0.1 |
| Arginine | no growth | no growth | 8.2 ± 0.1 |
| Serine | no growth | no growth | 3.8 ± 0 |
| Glycine | no growth | no growth | 5.1 ± 0 |
| Cysteine | no growth | no growth | 7.6 ± 0 |
| Tryptophan | 5.3 ± 0 | 3.1 ± 0.1 | 3.3 ± 0.1 |
| Tyrosine | 23.9 ± 2.5 | 14.3 ± 0 | no growth |
| Phenylalanine | no growth | 14.4 ± 0 | no growth |
