# Supplementary material for: On the flexibility of the cellular amination network in E coli
Source: eLife. 2022 Jul 25;11:e77492. doi: 10.7554/eLife.77492 (PMC9436414; doi:10.7554/eLife.77492)
Supplement: Supplementary file 2. — After sequencing the genomes of two independently isolated glut-aux + alaA mutants and the glut-aux + alaA parent, the results were mapped against the E. coli MG1655 reference genome (GenBank accession no. U00096.3) using Breseq. Mutations occurring in the mutants and not the parent are listed. [file elife-77492-supp2.docx]

| **Strain** | **position** | **mutation** | **annotation** | **gene** | **description** |
| --- | --- | --- | --- | --- | --- |
| Glutaux +alaA mutant 1 | 1,238,253 | (TATTGAGCGTATCTGGAGCGCGATCG)_1→2_ | coding (151/1071 nt) | *dadX* → | alanine racemase 2 |
| Glutaux +alaA mutant 2 | 1,238,923 | Δ1 bp | coding (821/1071 nt) | *dadX* → | alanine racemase 2 |
