# Supplementary material for: On the flexibility of the cellular amination network in E coli
Source: eLife. 2022 Jul 25;11:e77492. doi: 10.7554/eLife.77492 (PMC9436414; doi:10.7554/eLife.77492)
Supplement: Supplementary file 3. — Enzyme activity was determined in a dichlorophenolindophenole (DCPIP)-coupled assay with various substrate concentrations and 60 µg protein for measurements with d-alanine and 280 µg protein for measurements with glycine. Data are mean ± SE. [file elife-77492-supp3.docx]

|  | DadA | | |
| --- | --- | --- | --- |
| substrate | *k*_cat_ (s^-1^) | *K*_M_ (mM) | *k*_cat_/*K*_M_ (M^-1^ s^-1^) |
| d-alanine | 0.81 ± 0.05 | 1.26 ± 0.26 | 6.42 × 10^2^ |
| d-serine | 0.37 ± 0.02 | 64.77 ± 9.28 | 5.71× 10^0^ |
| glycine | 0.17 ± 0.08 | 617.40 ± 463.2 | 2.75 × 10^-1^ |
