# Supplementary material for: On the flexibility of the cellular amination network in E coli
Source: eLife. 2022 Jul 25;11:e77492. doi: 10.7554/eLife.77492 (PMC9436414; doi:10.7554/eLife.77492)
Supplement: Supplementary file 4. — Enzyme activity was determined in an assay coupling BhcA-dependent l-serine transamination to NADPH-dependent hydroxypyruvate reduction by GhrA with varying concentrations of l-serine or oxaloacetate for the respective activity measurements. Data are mean ± SE. [file elife-77492-supp4.docx]

|  | BhcA | | |
| --- | --- | --- | --- |
| substrate | *k*_cat_ (s^-1^) | *K*_M_ (mM) | *k*_cat_/*K*_M_ (M^-1^ s^-1^) |
| l-serine | 25.87 ± 0.4 | 7.01 ± 0.46 | 3.7 × 10^3^ |
| oxaloacetate | 31.23 ± 0.89 | 0.23 ± 0.03 | 1.4 × 10^5^ |
