# Supplementary material for: On the flexibility of the cellular amination network in E coli
Source: eLife. 2022 Jul 25;11:e77492. doi: 10.7554/eLife.77492 (PMC9436414; doi:10.7554/eLife.77492)
Supplement: Supplementary file 5. — ‘KO’ primers were used to amplify the knockout kanamycin (Km) cassette from pKD4 with 50 bp gene-specific upstream and downstream sequences. ‘KO-Ver’ primers (knockout verification) were used to verify gene replacement by Km resistance cassette and cassette removal by flippase. External and internal primers were used to verify successful removal of the gene from the genome. [file elife-77492-supp5.docx]

| name | Sequence (5’ 🡪 3’)) | purpose |
| --- | --- | --- |
| *gdhA*-KO-fwd | TAAACAACATAAGCACAATCGTATTAATATATAAGGGTTTTATATCTATGGTGTAGGCTGGAGCTGCTTC | Cassette amplification |
| *gdhA*-KO-rvs | TAAGCGTAGCGCCATCAGGCATTTACAACTTAAATCACACCCTGCGCCAGCATATGAATATCCTCCTTAG | Cassette amplification |
| *gdhA*-KO-Ver-fwd | CTGAGTTATCGCATTTGGTTATGAGATTACTCTCG | Gene removal verification |
| *gdhA*-KO-Ver-fwd | GGAGCATCATCCGTTAAATACTCATAAACGCCTG | Gene removal verification |
| *gdhA-*int-fwd | ATGCATgatcagacatattctctggag | Gene removal verification |
| *gdhA-*int-rvs | TCTAGAGCTAGCttaaatcacaccctgcgcc | Gene removal verification |
| *gltBD*-KO-fwd | GCGGTTCGGAAGTGGGGTTCCCGCAGAGCCTGGGGGAGGTTCACGATATGGTGTAGGCTGGAGCTGCTTC | Cassette amplification |
| *gltBD*-KO-rvs | ACAGTCTGGCGAATTCATTGTTACCTCGCTTAAACTTCCAGCCAGTTCATCATATGAATATCCTCCTTAG | Cassette amplification |
| *glttBD*-KO-Ver-fwd | GAGGCGCGCGTATGACACGCAAACC | Gene removal verification |
| *gltBD*-KO-Ver-rvs | GCACAATTTATTGAAAATTATCCCTATTATAGGAAAGGTCAAACG | Gene removal verification |
| *gltBD*-int-fwd | CTGTGCGACAAAGCCGAAAA | Gene removal verification |
| *gltBD*-int-rvs | TCGTTCAGAGTGCAGGAACC | Gene removal verification |
| *putA*-KO-Ver-fwd | GCTGGCGGCGATCAAAGA | Gene removal verification |
| *putA*-KO-Ver-rvs | GCGGTTGCACCTTTCAAAAATGTTAACTG | Gene removal verification |
| *putA*-int-fwd | gcagcggtattcacactcag | Gene removal verification |
| *putA*-int-rvs | cacaccagccattcctcgac | Gene removal verification |
| *ybdL*-KO-fwd | TCGTGTTATAGTGCCTTCAACACGCAACTTCGTCAGGTACAATAAAAATGGTGTAGGCTGGAGCTGCTTC | Cassette amplification |
| *ybdL*-KO-rvs | AATATTCCCGCGCCTGGACGGTTAAATAGCTAAAGCTGGCGCAGGCGTTCCATATGAATATCCTCCTTAG | Cassette amplification |
| *ybdL*-KO-Ver-fwd | CTGCAATGACCGCGAAACAA | Gene removal verification |
| *ybdL*-KO-Ver-rvs | TCGTGGACGCCATCAAATCA | Gene removal verification |
| *ybdL*-int-fwd | TTCCACAACTTGGCACCACT | Gene removal verification |
| *ybdL*-int-rvs | ATAACCCACTTTCCAGCCGG | Gene removal verification |
| *aspC*-KO-fwd | TTTTCAGCGGGCTTCATTGTTTTTAATGCTTACAGCACTGCCACAATCGCGTGTAGGCTGGAGCTGCTTC | Cassette amplification |
| *aspC*-KO- rvs | TACCCTGATAGCGGACTTCCCTTCTGTAACCATAATGGAACCTCGTCATGCATATGAATATCCTCCTTAG | Cassette amplification |
| *aspC*-KO-Ver-fwd | GCCTGCATAATCCCTTCCTGCA | Gene removal verification |
| *aspC*-KO-Ver-rvs | GTCTTGCAAAAACAGCCTGCGT | Gene removal verification |
| *aspC*-int-fwd | ATGCATtttgagaacattaccgccgc | Gene removal verification |
| *aspC*-int-rvs | GCTAGCTCTAGAttacagcactgccacaatcg | Gene removal verification |
| *dadX*-KO-Ver-fwd | ACTTTCTGGACTGGTCTGCG | Gene removal verification |
| *dadX*-KO-Ver-rvs | GGTTGCGATGCTTTGCTGAA | Gene removal verification |
| *dadX*-int-fwd | GATACAGGCCAGCCTCGATC | Gene removal verification |
| *dadX*-int-rvs | GGCAAGGCGTTAAATCGACC | Gene removal verification |
| *dadA*-KO-fwd | GATTAGATTATTATTCTTTTACTGTATCTACCGTTATCGGAGTGGCTATGGTGTAGGCTGGAGCTGCTTC | Cassette amplification |
| *dadA*-KO-rvs | GCCTGTATCGGACGGGTCATCTCGTTTCCTTAGCTGTGTGCGCCATGTAACATATGAATATCCTCCTTAG | Cassette amplification |
| *dadA*-KO-Ver-fwd | GACGCCATATTGCCGCAGAGTCAGG | Gene removal verification |
| *dadA*-KO-Ver-rvs | GTCGCGGCCTGGCGGACAATG | Gene removal verification |
| *dadA*-int-fwd | cgagttgtcatactgggaagtggtg | Gene removal verification |
| *dadA*-int-rvs | cattgggtaactgcaggccgc | Gene removal verification |
| *gcvTHP*-KO-fwd | CGCCTTTAGAAAATAGTCGAATCAGTGAATTACTGGTATTCGCTAATCGGAATTAACCCTCACTAAAGGGCG | Cassette amplification |
| *gcvTHP*-KO-rvs | AAGGAGAGAGGTTCACAATTCACTGCACGTTTCAGGAACCATCGCTCATGTAATACGACTCACTATAGGGCTC | Cassette amplification |
| *gcvTHP*-KO-Ver-fwd | TCTTCTGCGGGAGAGGATCA | Gene removal verification |
| *gcvTHP*-KO-Ver-rvs | ACCCTAACCCTCTCCCCAAA | Gene removal verification |
| *gcvTHP*-int-fwd | CAGCAGCACGTTGAAAAGCT | Gene removal verification |
| *gcvTHP*-int-rvs | TGGAAGCGGGCATGAATCTT | Gene removal verification |
| pZ-ASS-seq-fwd | GCATTTATCAGGGTTATTGTCTCATG | Amplification of pZ-ASS-insert |
| pZ-ASS-seq-rvs | CTAGGGCGGCGGATTTGTCCTAC | Amplification of pZ-ASS-insert |
| Cap-seq-rvs | CTGAACGGTCTGGTTATAGG | Amplification of pZ-ASS-insert |
| *alaA*-amp_fwd | CAATGCATCATCACCATCACCACTCCCCCATTGAAAAATCCAGCAAATTAGAGAATGTCTG | Amplification of *alaA* from genome |
| *alaA*-amp_rvs | GTGCTAGCTCTAGATTACAGCTGATGATAACCAGAAAGGAAACGCGC | Amplification of *alaA* from genome |
| *alaC*-amp_fwd | CAATGCATCATCACCATCACCACGCTGACACTCGCCCTGAACGTCG | Amplification of *alaC* from genome |
| *alaC*-amp_rvs | GTGCTAGCTCTAGATTATTCCGCGTTTTCGTGAATATGTTTGCTGCTG | Amplification of *alaC* from genome |
